# The effects of the Posterior X Taping versus augmented feedback on lower-extremity kinematic and muscle activity pattern during unilateral weight-bearing activities in men with tibiofemoral varus malalignment

**DOI:** 10.1186/s40634-023-00636-6

**Published:** 2023-07-20

**Authors:** Mohamadreza Hatefi, Malihe Hadadnezhad, Sadredin Shojaedin, Farideh Babakhani, Mehdi Khaleghi Tazji

**Affiliations:** 1 Department of Biomechanics and Sport Injuries, Faculty of Physical Education, Kharazmi University, Tehran, Iran; 2 Department of Sport Injuries and Corrective Exercises, Faculty of Physical Education, Allameh Tabataba’i University, Tehran, Iran

**Keywords:** Postural malalignment, Knee injury prevention, Single-legged squat, Forward step-down

## Abstract

**Purpose:**

Tibiofemoral Varus Malalignment (TFRV) contributes to overuse injuries by altering lower limb biomechanics. Both Posterior X Taping (PXT) and Real Time Feedback (RTF), have each been recommended for subjects with TFRV as they are thought to enhance control of excessive tibiofemoral rotations. This paper evaluates this claim.

**Methods:**

A total of recreational male 24 athletes with TFRV participated in the current study. Kinematic and electromyography variables of lower extremity were synchronously ​recorded on five consecutive repetitions of the single-legged-squat (SLS) and forward-step-down) FSD) tasks before and after applications of PXT and RTF.

**Results:**

The subjects at post-intervention in RTF group exhibited decreased hip adduction during FSD, and decreased hip adduction and internal rotation during eccentric and concentric phases of the SLS; Additionally, we observed increased gluteus medius activity during eccentric phase of the SLS and FSD tasks. In contrast, subjects at the post-intervention in PXT group exhibited decreased tibiofemoral external rotation and increased ankle external rotation during all the phases of both SLS and FSD tasks.

**Conclusion:**

These results suggest that the PXT and RTF interventions are recommended to immediately improve the functional defects of the subjects with TFRV during SLS and FSD tasks.

## Introduction

Functional activities begin with static postures, and because of the potential relationship between posture and movement, movement patterns probably be affected by lower extremity malalignments [1]. Tibiofemoral varus malalignment (TFRV) contributes to dysfunctional movement patterns during various activities and exposes subjects to overuse injuries, as one of the intrinsic risk factors. TFRV is primarily known as postural malalignment, which is caused by the combination of excessive internal hip rotation and knee hyperextension [2]; In this case, the lower extremity is recognized as a genu varum deformity in the static standing posture and dynamic knee valgus malalignment in dynamic activities that is accompanied by excessive hip adduction and medial rotation.

It is well established that biomechanical deficiencies including excessive adduction and internal rotation of the hip and tibiofemoral external rotation and, subsequently altered muscle activity patterns during functional activities can be associated with overuse injuries [3–7]. Thus, it is important to assess and correct the movement defects, otherwise, over time this can cause more malalignment, exacerbating the symptoms of osteoarthritis, increasing the risk of overuse injuries such as iliotibial band and patellofemoral pain syndromes, and leading to other problems [8–13]. Interestingly, other studies reported that many dysfunctional movement patterns can be improved by providing exercise interventions and corrective instructions [14].

Exercise-based intervention programs have been proposed for the management of TFRV [2]. Exercise aiming to strengthen the hip external rotator muscles and improve dysfunctional movement patterns, defined as uncontrolled movements that are visible during active movements [15], during functional tasks and activities of daily living are considered a priority [2]. Augmented feedback and kinesio tape have been recommended to improve biomechanical deficiencies in subjects with functional dysfunctions [16–20]. For individuals with TFTV, the posterior X taping (PXT) has been introduced aiming to control excessive tibiofemoral rotation during various activities as an effective method [2]; However, no evidence-based clinical research exists to support this argument yet. Park et al. [21] in a similar investigation demonstrated the effect of PXT on improvement in self-reported pain and knee stability in people with knee osteoarthritis during functional activities; Although they did not measure the lower extremity kinematics, but attributed this improvement to the effect of PXT on tibiofemoral rotation control. In contrast, Eui-hwan et al. [12] in a study demonstrated that no tibiofemoral rotation alterations have occurred as a result of the PXT intervention during the forward-step-down task; Despite, they detected reduction in knee pain and improving the scores of forward-step-down (FSD) performance. Notably, they used rigid tape and the subjects in their investigation were people with patellofemoral pain syndrome without considering the biomechanical deficiencies as one of the inclusion criteria.

On the other hand, evidence recommended using the augmented feedback aimed to improve the dysfunctional movement patterns during various activities as an effective intervention [22]. Among the various methods of providing feedback, visual real-time feedback (RTF) is used aiming to an immediate biomechanics deficiencies improvement as well as reinforce the learning of new movement patterns by neuromuscular alteration [22]; The RTF enables subjects to observe their movements and produce immediate biomechanical changes in their activity [23–25], which has been shown as an effective method to improve the performance of muscle when compared with post-task feedback. In relation to functional biomechanical deficiencies caused by muscles imbalance, there is an argument that isolated strengthening and stretching exercises may not cause the movement patterns improvement, but improving the functional biomechanical deficiencies during the dynamic activities could be accompanied by strengthening and stretching of the involved muscles at the optimal muscle length and adequate load which leading to neuromuscular adaptation [2].

**Fig. 1:**
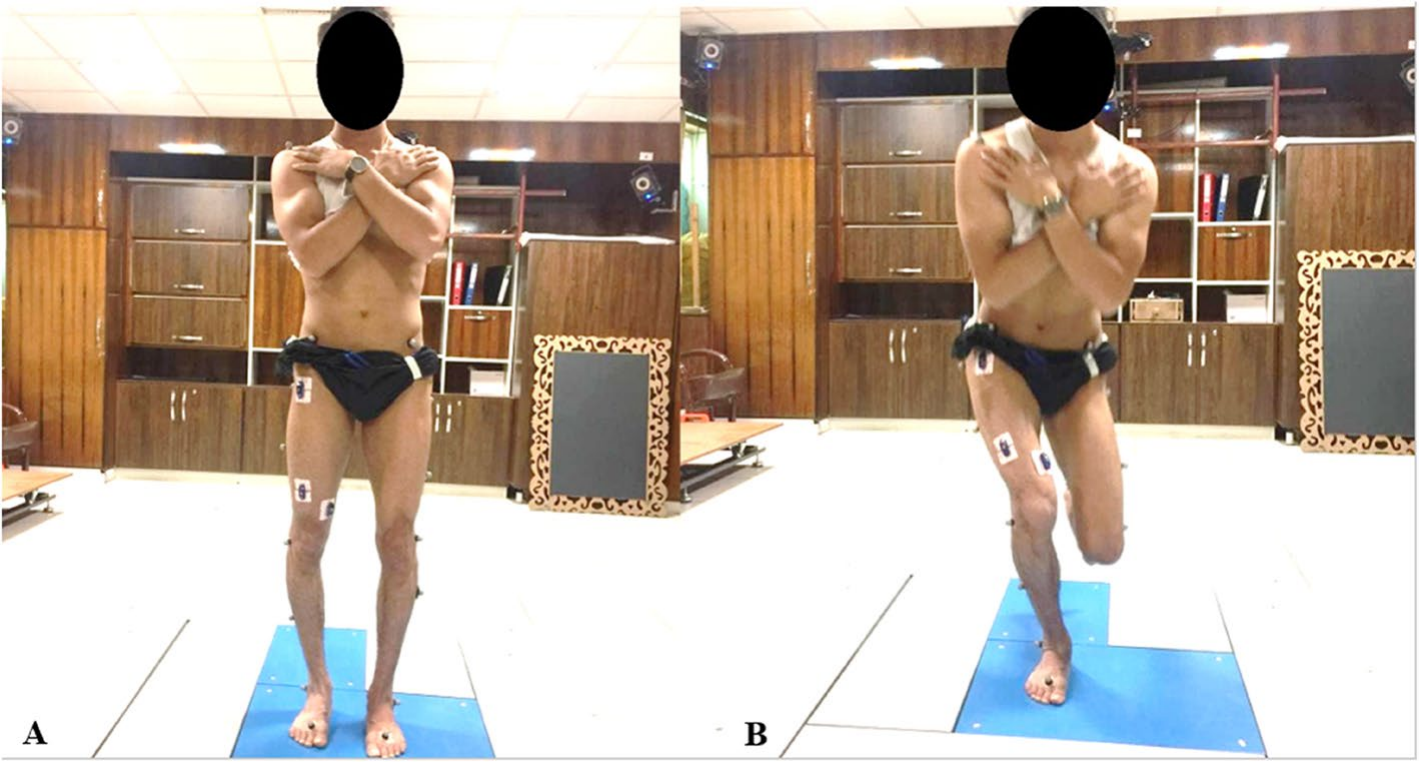
The subject exhibiting the genu varum deformity in the static position and excessive hip adduction and internal rotation during the single-leg squat task which was defines as TFRV

The purpose of the current research was to investigate and compare the effects of RTF and PXT interventions on lower extremity kinematic and muscles activity during the FSD and Single-legged squat (SLS) tasks in subjects with TFRV; Notably, we investigated the effect of the interventions on the functional alignment of the subjects with TFRV (Fig. 1).

## Materials and methods

### Study participants

This was a controlled laboratory study with a pre-and post-intervention trial. According to G. Power software version 3.1.0 (Franz Faul, University of Kiel, Germany), based using a repeated-measures ANOVA statistical test and assuming a power of 0.90, an effect size of 0.35, and an alpha level of 0.05, 24 recreational male athletes with TFRV were required for this study (Table 1); Which were selected according to the study criteria and then in a 1:1 ratio randomized to RTF and PXT groups using computer-generated number allocation; The allocation sequence was concealed from the researchers enrolling and assessing participants in sequentially numbered, opaque, and sealed envelopes. In this study, a recreational athlete was defined as a subject who participates in aerobic or sports activities at least three times a week for at least 30 min [22]. Inclusion criteria were: being a recreational athlete, aged 18 to 25 years, body mass index (BMI) between 18 and 24, normal ankle dorsiflexion range of motion at least 20° based on the ankle lunge test [26], the distance between the medial femoral condyles should be more than 3 cm in the standing with feet together position, and observed hip adduction and internal rotation in the functional tests (Single leg squat and step down tasks); which were defined as two parameters of TFRV (Fig. 1): evaluated by a specified corrective exercise-certificated specialist, and not participating in lower extremity rehabilitation programs in the last 6 months. Participants were excluded if they: had any musculoskeletal injury in the previous two months or lower-extremity injury in the previous six months, had a lower limb surgery or fractures within the past one year, and had any neurological and pathological conditions.

**Table 1:** Demographic characteristics of the subjects^^^

**Variables**	Groups		*p* value
	**RTF (*n* = 12)**	**PXT (*n* = 12)**	
**Age, yo**	25.61 ± 2.39	25.45 ± 1.27	.162
**Wight, kg**	73.18 ± 2.20	72.93 ± 3.45	.240
**Height, cm**	174.59 ± 3.68	175.48 ± 3.31	.411
**BMI, kg/m**^**2**^	23.89 ± .98	23.34 ± 1.19	.534
**Distance Between the Medial Femoral Condyles, cm**	4.21 ± 0.47	4.18 ± 0.41	.281
**Ankle Dorsiflexion, deg**	21.24 ± 1.81	21.08 ± 1.54	.187

^^^Data are presented as mean ± SD

*BMI* Body mass index

^*^Significant level: *p* ≤ .05

Prior to the test, ethical approval was obtained by the ethical committee of the sport sciences research institute of Iran (IR.SSRI.REC.1399.939), and all participants provided written informed consent.

### Procedures

In the present study, participants were referred to the Motion Analysis Laboratory on one occasion and completed a single 1-h testing session. They were asked to wear comfortable sports clothing without shoes aiming to prevent the influence of footwear differences. Overall, based on which group the participants were assigned, each of them performed the SLS and FSD tasks before and after PXT or RTF intervention with a 2-min rest period between tasks; Simultaneous data recordings of the kinematic (Vicon MX System; Oxford Metrics, UK) at sampling 250 Hz and electromyography (EMG, Myon m320RX, Schwarzenberg, Switzerland) at sampling 1000 Hz were performed on five consecutive repetitions of each trial, and then three intermediate consecutive repetitions were considered for analysis. Before the test, each participant performed a 5-min pedaling with level one resistance and 15 rpm speed as a warm-up. To perform the SLS task, first, participants were asked to stand on the dominant leg, defined as the preferred kicking leg, and flex the knee of the other leg to 90° and arms crossed on their chest. After that, they were asked to perform the five consecutive repetitions of the SLS task at a comfortable pace to a depth of approximately 60° of knee flexion while maintaining the trunk straight. To perform the FSD task, first, the participants were asked to stand on the step with the dominant leg, the non-weight-bearing leg straight and suspended in front of the step, and their hands on the pelvis. Similar to the SLS task, they were asked to perform five consecutive repetitions of the FSD task at a comfortable pace, bending the knee to the point that the opposite heel touched the floor without transmitting the weight, then returned to the initial position. The step height was adjusted for each of the participants so that the knee was positioned approximately at 30° flexion when the heel of the opposite leg touched the floor.

**Fig. 2:**
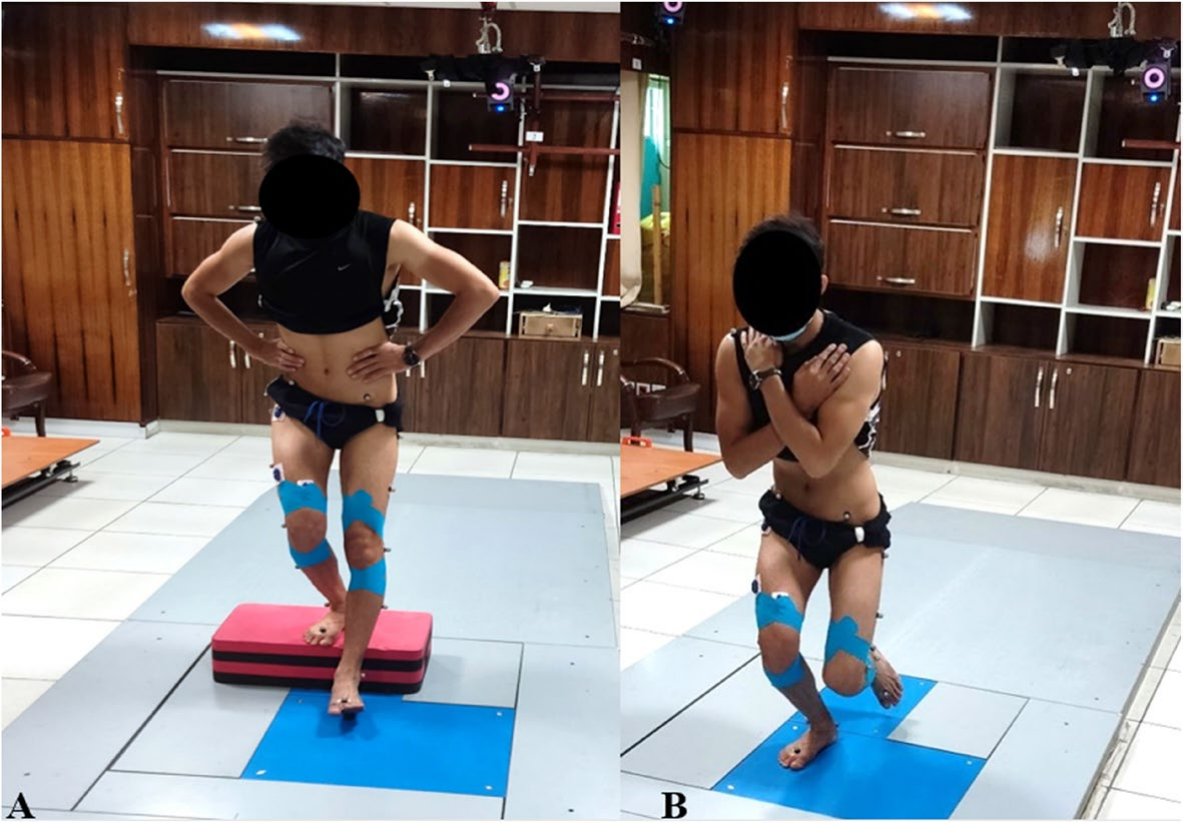
Performing the FSD (**A**) and SLS (**B**) tasks with PXT condition

In the PXT intervention, the elastic tape of 5 cm width (Kinesiology tape, MIKROS GmbH, Hamburg, Germany) was applied to the participants’ legs by an experienced corrective exercise specialist, while the participants were standing position with the knee flexed to 20° [12]. In the PXT technique, two strips with moderate intensity were applied in a spiral fashion on participants’ legs without causing discomfort. The first strip was applied from the lateral femur to the medial tibia aiming to control excessive tibiofemoral rotations [2]. The second strip was applied from the medial femur to the lateral tibia aiming to control knee hyperextension [2]. Notably, the origin and insertion of two strips were above the anterior femoral condyle and below the anterior tibial plateau respectively. In this technique, the stripes were symmetrically X-shaped from the posterior view of the knee (Fig. 2).

**Fig. 3:**
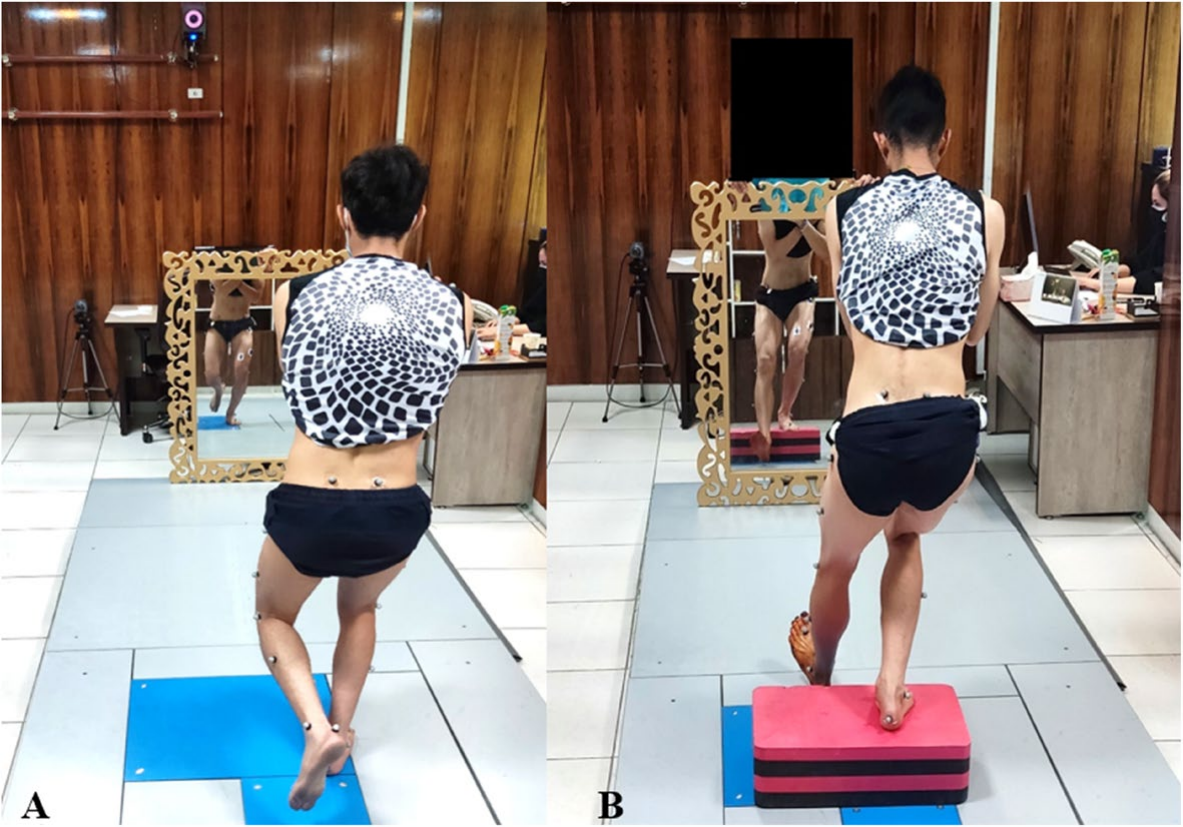
Performing the SLS (**A**) and FSD (**B**) tasks with RTF condition

In the RTF intervention, participants received external feedback regarding the lower extremity kinematic during each of the tasks via a full-length mirror placed in front of them. They were instructed by the corrective exercise specialist to keep their knees apart, patella facing forward, and prevent them from approaching the midline of the body, as a using external focus of attention; Notably, the timeframe between instruction and testing was less than a minute. No other feedback was given to the participants (Fig. 3).

### Electromyography measurement

According to the SENIAM recommendations [27], electrode locations for studied muscles were placed on the dominant leg in the muscle fibers direction, defined as the preferred kicking leg [28]. The gluteus medius (Gmed) electrode was placed at 50% on the line from the crista iliaca to the ipsilateral trochanter; the tensor fascia latae (TFL) electrode was placed at the line from the anterior spina iliaca superior to the lateral femoral condyle in the proximal 1/6; the vastus medialis (VM) electrode was placed at 80% on the line between the anterior spina iliaca superior and the joint space in front of the anterior border of the medial collateral ligament; and the vastus lateralis (VL) electrode was placed at 2/3 on the line from the anterior spina iliaca superior to the lateral side of the patella. Also, all electrode-applied locations were confirmed via isometric muscle contraction. Prior to placing the electrodes, the surface of the skin was shaved, abraded, and cleaned with isopropyl alcohol to reduce skin impedance.

Surface wireless electromyography was used to quantify the VM, VL, Gmed, and TFL activation. Raw EMG signals were recorded at the sampling rate of 1000 Hz, full-wave rectified, and then data noise was filtered at the 20–490 Hz band-pass and smoothed by the symmetrical moving RMS filter. RMS EMG activity (mean average amplitude) was calculated for three consecutive repetitions of each task before and after intervention in both RTF and PXT groups. The EMG data were normalized to the peak RMS EMG obtained during activity; Therefore, the average EMG data during each task were expressed as a percentage of the peak RMS EMG of each trial. It is noteworthy that the mean muscle activity during both the SLS and FSD tasks was calculated in two eccentric and concentric phases, separately; The eccentric phase was defined from the beginning of the knee flexion to maximum knee flexion; The concentric phase was defined from maximum knee flexion to maximum knee extension. Moreover, the Onset timing of the muscle activation also was calculated relative to the beginning of each task, eccentric phase, as well as the mean average amplitude of each muscle. Also, the VM:VL and Gmed:TFL activity ratio were calculated by dividing the normalized mean EMG of the VM to the normalized mean EMG of the VL, and the normalized mean EMG of the Gmed to the normalized mean EMG of the TFL, respectively. All EMG data were processed using MATLAB software (Mathworks, Natick, MA).

### Kinematic measurement

Prior to the data collection, eighteen reflective anatomical 15 mm-markers bilaterally were placed on each subject’s body according to the lower extremity model of the Plug-in Gait. In the next step, to determine the anatomical segment coordinate systems as a static calibration trial, subjects were asked to stand in a static reference position with feet placement shoulder-width apart.

Three-dimensional of hip, knee, and ankle joint angles measurements were captured by using a ten-camera motion analysis system at a sampling rate of 250 Hz in the all three movements planes during the FSD and SLS tasks for each trial. The mean joint angles of the three consecutive repetitions during the eccentric and concentric phase, and at maximum knee flexion were used for the analysis. All data processing including marker trajectories, signal processing, and obtaining three-dimensional kinematics was carried out using Vicon Nexus (version 2.5) and MATLAB (Mathworks, Natick, Massachusetts, USA) software.

### Statistical analysis

The normality of data distribution was confirmed by using the Shapiro–Wilk test; Thus, the 2 (Time main effect: pre-vs post-intervention) × 2 (Group main effect: PXT vs RTF) mixed-design analysis of variance (ANOVA) was used to compare the kinematic and muscles activity variables before and after intervention in each group (RTF and PXT) as well as intergroup comparisons, for each task separately. Moreover, if the main effects or time × group interaction were significant, the Bonferroni post hoc test was used for simple-effects testing. All data were calculated by use of the SPSS software Version 22.0 (Microsoft Corp., Redmond, WA), and the significance level was set at 0.05.

## Results

In the SLS task, the subjects at the post-intervention in the RTF group exhibited significantly less hip adduction (Difference = 6.476; *P* = 0.001 [95% CI, 3.111 to 9.841]) in the maximum knee flexion position. In contrast, in the PXT group, the subjects at the post-intervention exhibited significantly less tibiofemoral external rotation in the eccentric phase (Difference = -9.285; *P* = 0.001 [95% CI, -14.053 to -4.517]), concentric phase (Difference = -9.133; *P* = 0.001 [95% CI, -13.808 to -4.458]), and maximum knee flexion position (Difference = -10.644; *P* = 0.004 [95% CI, -17.183 to -4.106]) (Table 2). Moreover, in terms of muscle activation, the subjects exhibited significantly higher Gmed activity in the eccentric phase when the RTF intervention was applied (Difference = -0.077; *P* = 0.013 [95% CI, -0.136 to -0.019]). Notably, there was no difference in the amount of the mean activity or onset timing of other muscles after applying the RTF or PXT interventions (Table 3).

**Table 2:** Three-dimensional lower extremity kinematic during the single leg squat task before and after the Posterior X Taping (PXT) and Real-Time Feedback (RTF) interventions^^^

**Variables**			Groups				*p* value		
			**RTF**		**PXT**				
			Pre-intervention	Post-intervention	Pre-intervention	Post-intervention	Time Main Effect	Group Main Effect	Group × Time Interaction
**Hip, deg**	Eccentric	Sagittal	42.78 ± 4.48	45.87 ± 5.30	42.01 ± 5.49	43.35 ± 4.55	.110	.945	.079
		Frontal	4.27 ± 2.83	3.01 ± 4.97	4.49 ± 2.64	4.94 ± 4.84	.576	.572	.250
		Transverse	-1.98 ± 10.81	-4.12 ± 11.47	-2.22 ± 10.70	-2.34 ± 12.48	.137	.894	.177
	Concentric	Sagittal	46.89 ± 9.60	48.89 ± 8.40	44.37 ± 7.24	46.56 ± 7.25	.169	.536	.951
		Frontal	6.85 ± 3.34	4.66 ± 0.09	6.39 ± 2.97	7.15 ± 5.27	.383	.646	.087
		Transverse	-4.04 ± 10.66	-5.31 ± 10.41	-4.09 ± 9.86	-4.02 ± 12.29	.471	.910	.424
	Maximum knee flexion	Sagittal	73.49 ± 10.99	73.92 ± 8.35	74.18 ± 10.70	74.82 ± 9.52	.699	.871	.942
		Frontal	15.30 ± 4.41	8.82 ± 4.97^a^	16.13 ± 4.31	15.46 ± 6.23	.006^*^	.121	.020^*^
		Transverse	-5.14 ± 12.98	-7.38 ± 14.38	-5.98 ± 12.96	-6.20 ± 15.33	.320	.981	.410
**Knee, deg**	Eccentric	Sagittal	42.06 ± 3.11	46.34 ± 6.45	44.70 ± 4.22	45.92 ± 3.36	.085	.519	.320
		Frontal	4.59 ± 5.58	5.86 ± 7.79	3.48 ± 6.09	3.48 ± 6.75	.497	.590	.499
		Transverse	-6.41 ± 11.81	-2.00 ± 15.88	-6.70 ± 10.44	2.58 ± 12.17^a^	.001^*^	.733	.143
	Concentric	Sagittal	45.01 ± 6.66	44.90 ± 8.90	42.58 ± 4.61	44.49 ± 6.19	.567	.647	.521
		Frontal	2.66 ± 5.59	4.56 ± 7.33	2.47 ± 5.59	2.23 ± 6.71	.352	.687	.236
		Transverse	-5.28 ± 11.66	-1.81 ± 14.85	-6.12 ± 11.11	3.00 ± 13.09^a^	.001^*^	.753	.087
	Maximum knee flexion	Sagittal	82.78 ± 4.29	78.57 ± 3.00	84.75 ± 8.05	84.61 ± 5.30	.066	.134	.083
		Frontal	0.49 ± 8.05	2.59 ± 10.33	-1.34 ± 8.95	-0.77 ± 10.65	.344	.579	.584
		Transverse	1.76 ± 14.52	6.34 ± 16.73	-0.01 ± 12.24	10.63 ± 15.04^a^	.003^*^	.860	.181
**Ankle, deg**	Eccentric	Sagittal	24.69 ± 2.63	25.73 ± 4.39	24.27 ± 2.56	26.13 ± 2.22	.118	.994	.458
		Frontal	1.27 ± 4.41	2.88 ± 3.42	0.96 ± 3.65	3.72 ± 3.85^a^	.001^*^	.890	.109
		Transverse	-3.06 ± 15.03	-8.37 ± 13.00^a^	-2.19 ± 14.25	-12.80 ± 14.82^a^	.001^*^	.805	.038^*^
	Concentric	Sagittal	24.27 ± 2.58	23.43 ± 4.67	21.84 ± 1.90	23.99 ± 3.83	.390	.554	.062
		Frontal	1.22 ± 4.30	2.44 ± 2.86	0.55 ± 3.58	3.39 ± 3.80^a^	.001^*^	.937	.116
		Transverse	-3.02 ± 14.75	-7.24 ± 11.17	-0.86 ± 13.75	-11.80 ± 15.51^a^	.001^*^	.862	.052
	Maximum knee flexion	Sagittal	39.79 ± 3.03	36.71 ± 2.50^a^	39.42 ± 3.81	41.71 ± 4.75	.629	.175	.005^*^
		Frontal	5.57 ± 4.10	5.43 ± 3.35	5.05 ± 3.37	7.64 ± 4.57	.078	.660	.052
		Transverse	-15.72 ± 13.50	-16.30 ± 11.72	-15.12 ± 13.05	-24.01 ± 16.35^a^	.023^*^	.600	.041^*^

Sign convention ( ±): Sagittal, flexion ( +)/ extension (-); Frontal, adduction ( +)/ abduction (-); Transverse, internal ( +)/ external (-) rotation

^a^within-group difference

^b^between-group difference

^^^Data are presented as mean ± SD

^*^Significant level: *p* ≤ .05; Bonferroni post-hoc test

**Table 3:** Timing of muscle onset and mean activity during the single leg squat task before and after the Posterior X Taping (PXT) and Real-Time Feedback (RTF) interventions^^^

**Variables**		Groups				*p* value		
		**RTF**		**PXT**				
		Pre-intervention	Post-intervention	Pre-intervention	Post-intervention	Time Main Effect	Group Main Effect	Group × Time Interaction
**Mean muscle activity, % Peak RMS**								
**VM**	Eccentric	0.91 ± 0.16	0.97 ± 0.14	0.93 ± 0.12	0.95 ± 0.09	.122	.954	.540
	Concentric	1.23 ± 0.17	1.22 ± 0.15	1.18 ± 0.19	1.18 ± 0.20	.913	.650	.973
**VL**	Eccentric	0.94 ± 0.15	1.00 ± 0.12	0.95 ± 0.09	1.00 ± 0.09	.051	.831	.732
	Concentric	1.17 ± 0.18	1.14 ± 0.17	1.13 ± 0.21	1.10 ± 0.20	.112	.670	.898
**Gmed**	Eccentric	.077 ± 0.09	0.84 ± 0.12^a^	0.75 ± 0.07	0.80 ± 0.13	.005^*^	.554	.507
	Concentric	1.26 ± 0.06	1.20 ± 0.13	1.26 ± 0.06	1.26 ± 0.06	.277	.446	.384
**TFL**	Eccentric	1.01 ± 0.07	1.02 ± 0.06	1.04 ± 0.06	1.06 ± 0.07	.477	.288	.890
	Concentric	1.02 ± 0.16	1.01 ± 0.10	1.00 ± 0.14	0.97 ± 0.15	.443	.658	.810
**VM:VL Ratio**	Eccentric	0.97 ± 0.07	0.97 ± 0.05	0.97 ± 0.07	0.95 ± 0.08	.345	.696	.666
	Concentric	1.06 ± 0.07	1.08 ± 0.06	1.05 ± 0.07	1.09 ± 0.10	.132	.966	.658
**Gmed:TFL Ratio**	Eccentric	0.75 ± 0.08	0.82 ± 0.11	0.72 ± 0.05	0.75 ± 0.11	.057	.198	.546
	Concentric	1.27 ± 0.25	1.20 ± 0.19	1.29 ± 0.19	1.31 ± 0.21	.605	.526	.207
**Onset timing, ms**								
**VM**		124.87 ± 177.58	108.34 ± 184.79	135.62 ± 104.09	89.87 ± 84.07	.130	.517	.405
**VL**		221.87 ± 232.08	191.01 ± 200.05	190.37 ± 77.23	119.87 ± 92.67	.985	.190	.426
**Gmed**		132.75 ± 238.46	35.50 ± 54.80	85.12 ± 141.215	37.25 ± 59.22	.073	.715	.521
**TFL**		144.01 ± 176.68	30.62 ± 28.50	52.62 ± 31.62	88.01 ± 93.80	.256	.671	.070

*VM* Vastus medialis, *VL* Vastus lateralis, *Gmed* Gluteus medius, *TFL* Tensor fasciae latae

^a^within-group difference

^b^between-group difference

^^^Data are presented as mean ± SD

^*^Significant level: *p* ≤ .05; Bonferroni post-hoc test

In the FSD task, the subjects at the post-intervention in the RTF group exhibited significantly less hip adduction (eccentric: Difference = 2.864; *P* = 0.001 [95% CI, 1342 to 4.386], concentric: Difference = 2.157; *P* = 0.046 [95% CI, 0.046 to 4.268]), less internal rotation (eccentric: Difference = 3.789; *P* = 0.003 [95% CI, 1.472 to 6.106], concentric: Difference = 2.932; *P* = 0.038 [95% CI, 0.193 to 5.670]) in the eccentric and concentric phases, and less tibiofemoral external rotation (Difference = -4.721; *P* = 0.037 [95% CI, -9.133 to -0.329]). Similar to the SLS task, the subjects at the post-intervention in the PXT group exhibited significantly less tibiofemoral external rotation in the eccentric phase (Difference = -7.866; *P* = 0.002 [95% CI, -12.402 to -3.330]), concentric phase (Difference = -7.148; *P* = 0.004 [95% CI, -11.540 to -2.756]), and maximum knee flexion position (Difference = -8.591; *P* = 0.010 [95% CI, -14.730 to -2.452]) (Table 4). Again, in terms of muscle activation, the subjects exhibited significantly higher Gmed activity in the eccentric phase when the RTF intervention was applied (Difference = -0.125; *P* = 0.001 [95% CI, -0.191 to -0.058]) (Table 5). Interestingly, no between-group differences in the evaluated parameters were observed in pre-and post-intervention, similarly to the SLS task (*P* ≥ 0.05).

**Table 4:** Three-dimensional lower extremity kinematic during the forward step down task before and after the Posterior X Taping (PXT) and Real-Time Feedback (RTF) interventions^^^

**Variables**			Groups				*p* value		
			**RTF**		**PXT**				
			Pre-intervention	Post-intervention	Pre-intervention	Post-intervention	Time Main Effect	Group Main Effect	Group × Time Interaction
**Hip, deg**	Eccentric	Sagittal	35.02 ± 3.40	35.93 ± 6.32	34.55 ± 4.60	34.43 ± 6.27	.700	.694	.617
		Frontal	6.31 ± 3.70	3.44 ± 4.08^a^	5.39 ± 2.97	5.50 ± 2.34	.016^*^	.727	.010^*^
		Transverse	1.85 ± 11.28	-1.94 ± 11.30^a^	1.59 ± 10.73	1.53 ± 12.31	.025^*^	.780	.028^*^
	Concentric	Sagittal	34.90 ± 6.25	35.92 ± 7.28	37.25 ± 7.55	34.39 ± 8.89	.511	.908	.173
		Frontal	6.99 ± 4.43	4.83 ± 3.51^a^	7.16 ± 3.92	5.45 ± 2.19	.015^*^	.815	.750
		Transverse	0.43 ± 11.79	-2.50 ± 11.01^a^	0.48 ± 11.12	-0.58 ± 11.50	.044^*^	.863	.318
	Maximum knee flexion	Sagittal	53.46 ± 9.44	56.50 ± 11.87	54.20 ± 12.33	56.10 ± 13.22	.092	.989	.720
		Frontal	14.52 ± 14.17	11.73 ± 4.63	13.75 ± 4.71	13.75 ± 3.32	.134	.752	.136
		Transverse	0.95 ± 15.50	-1.72 ± 14.67	1.12 ± 14.05	-0.02 ± 17.56	.093	.905	.482
**Knee, deg**	Eccentric	Sagittal	38.85 ± 3.87	40.83 ± 7.71	38.54 ± 7.63	40.06 ± 7.64	.224	.991	.471
		Frontal	6.17 ± 7.84	7.21 ± 8.93	6.53 ± 7.53	7.67 ± 8.95	.500	.983	.606
		Transverse	-7.86 ± 11.35	-3.36 ± 14.45	-7.61 ± 12.22	0.25 ± 13.07^a^	.001^*^	.762	.280
	Concentric	Sagittal	36.17 ± 6.93	38.21 ± 7.66	40.53 ± 8.55	35.61 ± 9.61	.426	.818	.067
		Frontal	5.44 ± 7.87	6.63 ± 8.22	5.59 ± 8.43	5.50 ± 8.45	.531	.905	.464
		Transverse	-8.43 ± 11.05	-3.72 ± 14.78^a^	-7.47 ± 11.68	-0.32 ± 12.37^a^	.001^*^	.726	.416
	Maximum knee flexion	Sagittal	66.45 ± 10.32	69.27 ± 11.29	68.52 ± 13.35	71.86 ± 13.17^a^	.013^*^	.702	.815
		Frontal	3.53 ± 13.08	5.99 ± 13.14	3.80 ± 12.17	4.12 ± 14.28	.231	.904	.348
		Transverse	-1.29 ± 13.06	4.11 ± 15.61	-0.93 ± 12.39	7.66 ± 14.99^a^	.004^*^	.777	.444
**Ankle, deg**	Eccentric	Sagittal	22.99 ± 3.96	24.50 ± 3.49	22.48 ± 4.34	24.50 ± 5.12	.093	.902	.608
		Frontal	0.97 ± 4.19	1.59 ± 3.28	0.62 ± 4.11	3.75 ± 3.80^a^	.001^*^	.643	.001^*^
		Transverse	-2.23 ± 14.63	-4.46 ± 12.28	-1.28 ± 16.18	-12.87 ± 15.45^a^	.001^*^	.617	.001^*^
	Concentric	Sagittal	20.74 ± 3.42	22.64 ± 3.77	22.02 ± 4.27	21.14 ± 3.60	.476	.951	.065
		Frontal	0.36 ± 4.01	1.56 ± 3.53^a^	0.62 ± 3.53	3.10 ± 3.18^a^	.001^*^	.616	.061
		Transverse	-0.48 ± 14.02	-4.17 ± 13.05^a^	-1.31 ± 14.01	-10.99 ± 13.62^a^	.001^*^	.581	.014^*^
	Maximum knee flexion	Sagittal	34.51 ± 4.66	35.98 ± 2.93	34.28 ± 3.90	38.57 ± 5.20^a^	.008^*^	.549	.152
		Frontal	4.38 ± 4.26	4.88 ± 3.46	4.01 ± 3.39	7.05 ± 4.16^a^	.010^*^	.628	.053
		Transverse	-12.25 ± 14.32	-12.93 ± 12.54	-12.14 ± 13.03	-22.71 ± 15.63^a^	.003^*^	.577	.050^*^

Sign convention ( ±): Sagittal, flexion ( +)/ extension (-); Frontal, adduction ( +)/ abduction (-); Transverse, internal ( +)/ external (-) rotation

^a^within-group difference

^b^between-group difference

^^^Data are presented as mean ± SD

^*^Significant level: *p* ≤ .05; Bonferroni post-hoc test

**Table 5:** Timing of muscle onset and mean activity during the forward step down task before and after the Posterior X Taping (PXT) and Real-Time Feedback (RTF) interventions^^^

**Variables**		Groups				*p* value		
		**RTF**		**PXT**				
		Pre-intervention	Post-intervention	Pre-intervention	Post-intervention	Time Main Effect	Group Main Effect	Group × Time Interaction
**Mean muscle activity, % Peak RMS**								
**VM**	Eccentric	0.92 ± 0.17	0.96 ± 0.12	0.91 ± 0.17	0.95 ± 0.17	.068	.934	.968
	Concentric	1.18 ± 0.10	1.17 ± 0.20	1.17 ± 0.16	1.12 ± 0.14	.141	.768	.056
**VL**	Eccentric	0.96 ± 0.17	0.98 ± 0.11	0.94 ± 0.19	0.97 ± 0.16	.442	.866	.855
	Concentric	1.11 ± 0.10	1.10 ± 0.18	1.12 ± 0.11	1.06 ± 0.11	.216	.385	.345
**Gmed**	Eccentric	0.70 ± 0.9	0.82 ± 0.14^a^	0.70 ± 0.08	0.75 ± 0.06	.001^*^	.394	.131
	Concentric	1.16 ± 0.13	1.22 ± 0.12	1.25 ± 0.18	1.23 ± 0.14	.729	.257	.521
**TFL**	Eccentric	1.02 ± 0.10	1.02 ± 0.09	1.01 ± 0.11	1.03 ± 0.09	.621	.882	.728
	Concentric	1.06 ± 0.13	1.03 ± 0.10	1.13 ± 0.14	1.04 ± 0.09	.108	.441	.361
**VM:VL Ratio**	Eccentric	0.95 ± 0.07	0.98 ± 0.07	0.97 ± 0.07	0.98 ± 0.06	.213	.819	.491
	Concentric	1.07 ± 0.04	1.06 ± 0.03	1.01 ± 0.06	1.05 ± 0.05	.209	.163	.057
**Gmed:TFL Ratio**	Eccentric	0.69 ± 0.10	0.80 ± 0.10^a^	0.70 ± 0.09	0.73 ± 0.08	**.013**^**a**^	.449	.170
	Concentric	1.11 ± 0.21	1.18 ± 0.13	1.11 ± 0.17	1.20 ± 0.16	.219	.843	.894
**Onset timing, ms**								
**VM**		78.87 ± 76.35	116.80 ± 228.15	104.50 ± 114.93	120.12 ± 134.78	.621	.787	.836
**VL**		101.75 ± 101.27	131.50 ± 138.69	157.50 ± 198.78	126.87 ± 104.14	.992	.647	.511
**Gmed**		92.63 ± 185.54	22.12 ± 23.02	126.37 ± 266.03	31.37 ± 34.11	.132	.739	.816
**TFL**		53.75 ± 53.44	134.62 ± 108.50	117.01 ± 134.80	116.50 ± 89.27	.073	.632	.070

*VM* Vastus medialis, *VL* Vastus lateralis, *Gmed* Gluteus medius, *TFL* Tensor fasciae latae

^a^within-group difference

^b^between-group difference

^^^Data are presented as mean ± SD

^*^Significant level: *p* ≤ .05; Bonferroni post-hoc test

## Discussion

This is the first study to investigate the effect of the PXT technique as well as RTF intervention on three-dimensional hip, knee, and ankle joint angles and muscle activation patterns. The results of the current study support our hypothesis that the PXT and RTF interventions would significantly change the lower extremity function of the subjects with TFRV during unilateral weight-bearing; We have shown that the tibiofemoral external rotation angle was decreased in all phases of the SLS and FSD tasks when the PXT intervention was applied; In contrast, the subjects at the post-intervention in the RTF group exhibited a decreased hip adduction and internal rotation during the eccentric and concentric phases of the FSD, and a decreased hip adduction during the SLS at the maximum knee flexion. Interestingly, no statistically significant difference was observed between groups. These results suggested that both interventions improve the lower extremity kinematics of the subjects with TRFV during the SLS and FSD task, as we hypothesized.

Based on the results of the current study, we confirm the hypothesis of the previous researchers that the effect of the PXT technique on controlling the excessive tibiofemoral external rotations during weight-bearing activities. Regarding the decreased excessive tibiofemoral external rotations at the PXT post-intervention, this intervention can be used as a knee osteoarthritis prevention approach in subjects with TFRV through the reduction of the internal and external knee compressive forces [21, 29], which would likely have clinical importance. On the other hand, it has been shown that there is a negative correlation between knee pain and decreased tibiofemoral internal rotation [30]; In addition to decreased tibiofemoral external rotation, we observed an increased ankle external rotation during all phases of the SLS and FSD tasks at the post-intervention in the PXT group. These results can be interpreted as an increase in the tibiofemoral internal rotation relative to its distal and proximal joints; We detected exactly this situation in the current study: increasing the tibial medial rotation relative to the femur and ankle during the tasks. Furthermore, despite decreased hip adduction and internal rotation after providing the PXT intervention, it was not statistically significant in any of the phases of the SLS and FSD tasks. Also, we did not observe any alterations in activity and onset timing of the muscles when the PXT intervention was applied.

In terms of the RTF intervention, the results of the current study are consistent with many similar studies, which suggest the RTF intervention immediately alters lower extremity kinematic parameters during various activities and in people with different conditions [22, 23, 31, 32]. As a clinical recommendation, given the importance of controlling hip adduction and internal rotation as biomechanical risk factors [5, 33, 34], providing the RTF intervention by improving lower extremity kinematics may prevent overuse injuries such as patellofemoral pain syndrome by decrease external force [16] in subjects with TFRV. In addition to kinematics, we observed an increased Gmed activity during the eccentric phase of both SLS and FSD tasks when the RTF intervention was applied, which is consistent with the kinematic results of the current study that the decreased hip adduction and internal rotation. Notably, when designing electromyography data-based exercises to increase the activity of a specific muscle, we have to consider its synergist muscles as well; Synergist muscles work together and affect each other during movement. It is well established that altered muscle activity patterns can contribute the overuse injuries by altered biomechanics and movement patterns [35–38]. In the current study, an increased Gmed:TFL ratio activity was observed during the eccentric phase of the FSD task at the post-intervention in the RTF group, interestingly, where decreased hip adduction and internal rotation were observed. Given these results, providing RTF intervention can change lower extremity kinematics and increase the Gmed activity at the appropriate hip position in the subjects with TFRV. In contrast, despite increased Gmed:TFL activity ratio during the eccentric phase of the SLS task, it was not statistically significant. The difference between the SLS and FSD tasks relative to Gmed:TFL activity ratio at the post-intervention in the RTF group can be explained by the kinematic results of the current study: Greater hip adduction and internal rotation control during the eccentric phase of the FSD than SLS.

Some studies with injury prevention and rehabilitation approaches have examined the effect of different interventions on increasing the VM to VL activity ratio [39–41]. Overall, most of these studies fall into two paradigms: first, these muscles do not increase relative to each other because both are innervated by the femoral nerve, thus increasing one muscle more than the other is not possible [4, 42]; Second, that several VM muscle fibers originated from hip adductor muscles, so stretching the adductor muscles when the hip is positioned in the abduction and external rotation increase the VM muscle length as well as its activity [43]. Eventually, the results of the current study were consistent with the studies of the first hypothesis that increasing a specific muscle activity than the others, innervated by the same nerve, is not possible; Although, based on the studies of the second hypothesis, it seems that the lack of altered muscle activity in the current study may be attributed to the lack of significant change in the hip position.

Interestingly, there was no statistical between-groups difference in muscle activity as in kinematics; This lack of between-groups differences may be due to the fact that both the RTF and PXT interventions in some way affect the lower extremity kinematics parameters. Nevertheless, for a comprehensive understanding of the PXT and RTF interventions' effect on lower extremity functions as an evidence-based clinical guideline for the management of TFRV malalignment, new studies must be conducted to evaluate the long-term effect of the interventions, retention of movement-pattern alterations, and the concept of ability to skill transfer like a one-legged landing from a jump.

This study had several limitations; First, this was a cross-sectional study, so its long-term effects are unclear, also we do not know if combining the RTF and PXT would improve these findings; Second, we recommend using the PXT to reduce tibiofemoral compressive loads as a result of controlling excessive tibiofemoral rotations during the SLS and FSD tasks based on the previous studies, therefore, future kinetics data-based studies are required to confirm this argument; Third, the participants in this study were male recreational athletes, so these results may not be generalizable to everyone.

## Conclusion

In summary, we investigated the effects of the PXT and RTF interventions on lower extremity kinematic and muscles activity during the SLS and FSD tasks; These results suggest that both interventions immediately change the kinematics in males with TFRV during the SLS and FSD tasks; Notably, as an evidence-based clinical intervention, the PXT technique can benefit the clinical environment by controlling excessive tibiofemoral rotation during unilateral weight-bearing activities for preventing overuse injuries.
